# 5-Chloro-2-methoxy­anilinium nitrate

**DOI:** 10.1107/S1600536808008805

**Published:** 2008-04-04

**Authors:** Hanene Hemissi, Sonia Abid, Mohamed Rzaigui

**Affiliations:** a Laboratoire de Chimie des Matériaux, Faculté des Sciences de Bizerte, 7021 Zarzouna Bizerte, Tunisia

## Abstract

The title salt, C_7_H_9_ClNO^+^·NO_3_^−^, exhibits extensive hydrogen bonding between the ammonium functional group and the nitrate anion. A two-dimensional network of bifurcated N—H⋯O hydrogen bonds generates corrugated layers in the *bc* plane. The organic mol­ecules are stacked in a parallel orientation as a result of π–π inter­actions, with an inter-ring distance of 3.837 Å.

## Related literature

For related literature, see: Abid *et al.* (2007 ▶); Aloui *et al.* (2002 ▶); Desiraju & Steiner (1999 ▶); Hemissi *et al.* (2005 ▶); Jayaraman *et al.* (2002 ▶); Ouslati & Ben Nasr (2006 ▶); Steiner (2002 ▶); Kefi *et al.* (2007 ▶).
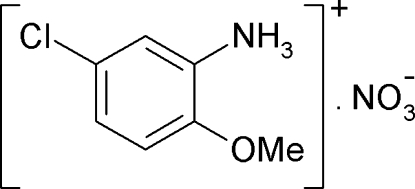


## Experimental

### 

#### Crystal data

C_7_H_9_ClNO^+^·NO_3_^−^
*M* *_r_* = 220.61
Monoclinic, 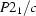

*a* = 10.681 (2) Å
*b* = 9.474 (3) Å
*c* = 9.802 (3) Å
β = 102.38 (3)°
*V* = 968.8 (5) Å^3^
*Z* = 4
Mo *K*α radiation
μ = 0.39 mm^−1^
*T* = 293 (2) K
0.2 × 0.18 × 0.16 mm

#### Data collection

Enraf–Nonius TurboCAD-4 diffractometer
Absorption correction: none
4244 measured reflections
2125 independent reflections
1409 reflections with *I* > 2σ(*I*)
*R*_int_ = 0.029
2 standard reflections frequency: 120 min intensity decay: 5%

#### Refinement

*R*[*F* ^2^ > 2σ(*F* ^2^)] = 0.041
*wR*(*F* ^2^) = 0.109
*S* = 1.02
2125 reflections
129 parameters
H-atom parameters constrained
Δρ_max_ = 0.18 e Å^−3^
Δρ_min_ = −0.42 e Å^−3^

### 

Data collection: *CAD-4 EXPRESS* (Enraf–Nonius, 1994 ▶); cell refinement: *CAD-4 EXPRESS*; data reduction: *XCAD4* (Harms & Wocadlo, 1995 ▶); program(s) used to solve structure: *SHELXS97* (Sheldrick, 2008 ▶); program(s) used to refine structure: *SHELXL97* (Sheldrick, 2008 ▶); molecular graphics: *ORTEP-3 for Windows* (Farrugia, 1997 ▶), *DIAMOND* (Brandenburg, 1998 ▶); software used to prepare material for publication: *WinGX* (Farrugia, 1999 ▶).

## Supplementary Material

Crystal structure: contains datablocks I, global. DOI: 10.1107/S1600536808008805/fj2102sup1.cif

Structure factors: contains datablocks I. DOI: 10.1107/S1600536808008805/fj2102Isup2.hkl

Additional supplementary materials:  crystallographic information; 3D view; checkCIF report

## Figures and Tables

**Table 1:** Hydrogen-bond geometry (Å, °)

*D*—H⋯*A*	*D*—H	H⋯*A*	*D*⋯*A*	*D*—H⋯*A*
N2—H1⋯O1^i^	0.89	2.08	2.967 (3)	173
N2—H1⋯O2^i^	0.89	2.57	3.103 (3)	120
N2—H2⋯O1	0.89	2.04	2.927 (3)	171
N2—H2⋯O3	0.89	2.38	3.043 (3)	131
N2—H3⋯O2^ii^	0.89	2.55	3.240 (3)	135
N2—H3⋯O3^ii^	0.89	2.07	2.939 (3)	165
C7—H9⋯O2^iii^	0.96	2.42	3.361 (4)	167

Symmetry codes: (i) 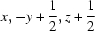; (ii) 
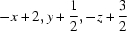
; (iii) 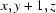.

**Fig. 1.**
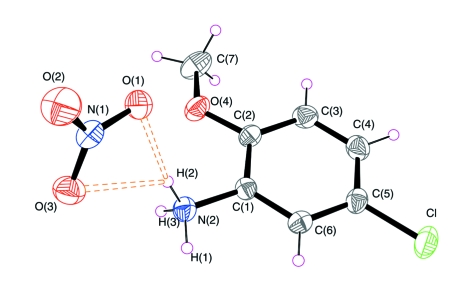
ORTEP-3 (Farrugia,(1999)) view of the title compound, with the atom numbering scheme. Displacement ellipsoids for non-H atoms are drawn at the 30% probability level, and H-atoms are shown as spheres with an arbitary radius.

**Fig. 2.**
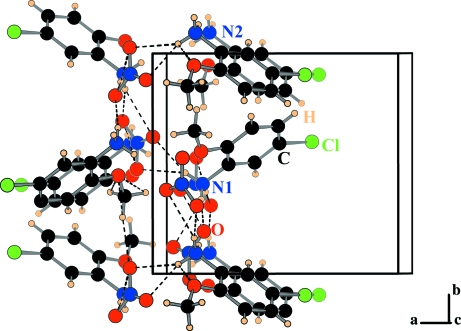
A perspective view of the atomic arrangement of the title compound.

**Fig. 3.**
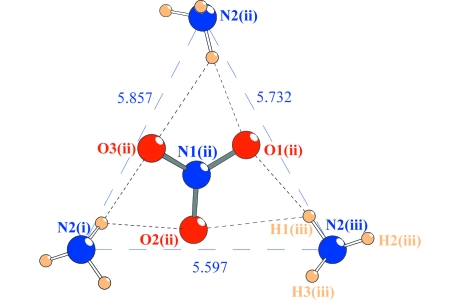
Nitrate anion environment in the title compound. [Symmetry operators: (i) x, y, z; (ii) -x, y + 1/2, -z + 1/2; (iii) -x, -y, -z].

## supplementary crystallographic information

### Comment

Hydrogen bonding is of intense interest because of their widespread occurrence in biological systems. So, it is very helpful to search simple molecules allowing to understanding the configuration and the function of some complex macromolecules.The hybrid compounds are rich in H-bonds and they could be used to this effect because of their potential importance in constructing sophisticated assemblies from discrete ionic or molecular building blocks due to its strength and directionality (Steiner. *et al.* 2002, Jayaraman, *et al.* 2002). In this work, the combination of 2-methoxy-5-chloroaniline and nitric acid has been chosen to elaborate the special hydrogen-bond pattern. The asymmetric unit of crystal structure, depicted in *ORTEP* drawing (Fig. 1), correspond to the formula unit C_6_H_9_ClNO^+^.NO_3_^-^. The main feature of this atomic arrangement is the existence of thick inorganic corrugated layers spreading around *bc* plane (Fig. 2). Inside layer, each NO_3_^-^ anion is linked to three organic groups through bifurcated N–H···O H-bonding. The N···O and H···O bond lengths are in the ranges of 2.927 (3)–3.240 (3) Å and 2.04–2.57 Å, respectively. The organic species interact also with a weak C–H···O hydrogen bond with H···O separation of 2.42 Å. These interactions are weaker than those observed otherwise (Ouslati *et al.* 2006, Kefi *et al.* 2007). The orientation of molecules in this framework is governed by a nearly regular triangular arrangement of 2-methoxy-5-chlorophenylammonium groups [N···N distances between nitrogen ammonium atoms are in the range of 5.597 and 5.857 Å, N···N···N angles range from 57.75 to 62.25°] (Fig. 3). As well as electrostatic and van der Waals forces and hydrogen bonds, aromatic π-π stacking interactions participate to define the crystal packing. Indeed, in the atomic arrangement of the title compound, the phenyl ring of the organic molecules are stacked in the parallel orientation with inter-planar separation of 3.837 Å indicating there are π–π stacking interactions (Desiraju & Steiner, 1999). The –Cl, –NH_3_ and –OCH_3_ substituents form, respectively, different torsion angles with the benzene ring: Cl—C5—C6—C1 ((t1) = 178.71°), N2—C1—C2—C3 ((t2) = -179.74°) and C7—O4—C2—C3 ((t3) = 12.43°). (t1) and (t2) angle values show that the chloro and amino substituents are nearly coplanar with the aryl ring. The torsion angle (t3) value indicates that the methoxy group lies out the plane of the benzene ring. This conformation resembles that observed in other compounds (Abid *et al.* 2007, Aloui *et al.* 2002, Hemissi *et al.* 2005).

### Experimental

An ethanolic 2-methoxy-5-chloroaniline solution (5 mmol, in 5 ml) was added to an aqueous HNO_3_ solution (0.5 *M*). The obtained solution is evaporated during several days in ambient condition until the formation of single crystals of the title compound.

### Refinement

All H atoms were positioned geometrically and treated as riding on their parent atoms, [N–H = 0.89, C–H =0.96 Å (CH_3 _) with with *U*_iso_(H) = 1.5Ueq and C–H =0.96 Å (Ar–H), with *U*_iso_(H) = 1.5Ueq]

### Crystal data

**Table:**

C_7_H_9_ClNO^+^·NO_3_^–^	*F*_000_ = 456
*M**_r_* = 220.61	*D*_x_ = 1.513 Mg m^−^^3^
Monoclinic, *P*2_1_/*c*	Mo *K*α radiation λ = 0.71073 Å
Hall symbol: -P 2ybc	Cell parameters from 25 reflections
*a* = 10.681 (2) Å	θ = 9.1–10.8º
*b* = 9.474 (3) Å	µ = 0.39 mm^−^^1^
*c* = 9.802 (3) Å	*T* = 293 (2) K
β = 102.38 (3)º	Prism, black
*V* = 968.8 (5) Å^3^	0.2 × 0.18 × 0.16 mm
*Z* = 4	

### Data collection

**Table:**

Enraf–Nonius TurboCAD4 diffractometer	θ_max_ = 27.0º
Monochromator: graphite	θ_min_ = 2.9º
non–profiled ω scans	*h* = −13→13
Absorption correction: none	*k* = −12→0
4244 measured reflections	*l* = −12→12
2125 independent reflections	2 standard reflections
1409 reflections with *I* > 2σ(*I*)	every 120 min
*R*_int_ = 0.029	intensity decay: 5%

### Refinement

**Table:**

Refinement on *F*^2^	Secondary atom site location: difference Fourier map
Least-squares matrix: full	Hydrogen site location: inferred from neighbouring sites
*R*[*F*^2^ > 2σ(*F*^2^)] = 0.041	H-atom parameters constrained
*wR*(*F*^2^) = 0.109	*w* = 1/[σ^2^(*F*_o_^2^) + (0.0495*P*)^2^ + 0.2112*P*] where *P* = (*F*_o_^2^ + 2*F*_c_^2^)/3
*S* = 1.02	(Δ/σ)_max_ < 0.001
2125 reflections	Δρ_max_ = 0.18 e Å^−^^3^
129 parameters	Δρ_min_ = −0.42 e Å^−^^3^
Primary atom site location: structure-invariant direct methods	Extinction correction: none

### Special details

**Table:**

Geometry. All e.s.d.'s (except the e.s.d. in the dihedral angle between two l.s. planes) are estimated using the full covariance matrix. The cell e.s.d.'s are taken into account individually in the estimation of e.s.d.'s in distances, angles and torsion angles; correlations between e.s.d.'s in cell parameters are only used when they are defined by crystal symmetry. An approximate (isotropic) treatment of cell e.s.d.'s is used for estimating e.s.d.'s involving l.s. planes.
Refinement. Refinement of *F*^2^ against ALL reflections. The weighted *R*-factor *wR* and goodness of fit *S* are based on *F*^2^, conventional *R*-factors *R* are based on *F*, with *F* set to zero for negative *F*^2^. The threshold expression of *F*^2^ > σ(*F*^2^) is used only for calculating *R*-factors(gt) *etc*. and is not relevant to the choice of reflections for refinement. *R*-factors based on *F*^2^ are statistically about twice as large as those based on *F*, and *R*- factors based on ALL data will be even larger.

### Fractional atomic coordinates and isotropic or equivalent isotropic displacement parameters (Å^2^)

**Table:**

	*x*	*y*	*z*	*U*_iso_*/*U*_eq_	
Cl	0.39657 (5)	0.59783 (8)	0.79445 (7)	0.0703 (2)	
N2	0.83926 (16)	0.40697 (19)	0.75239 (18)	0.0478 (4)	
H2	0.8415	0.3375	0.6918	0.072*	
H1	0.8331	0.3705	0.8344	0.072*	
H3	0.9107	0.4579	0.7630	0.072*	
O4	0.83237 (15)	0.55493 (16)	0.52279 (16)	0.0568 (4)	
C1	0.72828 (18)	0.4973 (2)	0.6998 (2)	0.0387 (4)	
C2	0.72905 (19)	0.5751 (2)	0.5790 (2)	0.0425 (5)	
C3	0.6254 (2)	0.6618 (2)	0.5276 (2)	0.0530 (6)	
H4	0.6241	0.7158	0.4481	0.064*	
C4	0.5237 (2)	0.6682 (2)	0.5941 (2)	0.0532 (5)	
H5	0.4537	0.7256	0.5585	0.064*	
C5	0.52562 (19)	0.5906 (2)	0.7117 (2)	0.0465 (5)	
C6	0.62876 (18)	0.5041 (2)	0.7675 (2)	0.0425 (5)	
H6	0.6303	0.4523	0.8484	0.051*	
C7	0.8528 (3)	0.6480 (3)	0.4147 (3)	0.0714 (7)	
H7	0.7883	0.6316	0.3316	0.107*	
H8	0.9360	0.6309	0.3957	0.107*	
H9	0.8477	0.7441	0.4443	0.107*	
O1	0.82176 (15)	0.19146 (16)	0.53507 (16)	0.0556 (4)	
N1	0.88696 (17)	0.09358 (19)	0.60238 (19)	0.0484 (4)	
O2	0.8812 (2)	−0.02617 (18)	0.5536 (2)	0.0835 (6)	
O3	0.95687 (16)	0.12025 (18)	0.71720 (17)	0.0648 (5)	

### Atomic displacement parameters (Å^2^)

**Table:**

	*U*^11^	*U*^22^	*U*^33^	*U*^12^	*U*^13^	*U*^23^
Cl	0.0462 (3)	0.0888 (5)	0.0816 (5)	−0.0011 (3)	0.0262 (3)	−0.0182 (4)
N2	0.0487 (9)	0.0465 (10)	0.0493 (10)	0.0074 (8)	0.0128 (8)	0.0016 (8)
O4	0.0608 (9)	0.0616 (10)	0.0553 (9)	0.0042 (8)	0.0284 (8)	0.0059 (8)
C1	0.0394 (9)	0.0342 (10)	0.0416 (10)	0.0017 (8)	0.0067 (8)	−0.0040 (8)
C2	0.0474 (11)	0.0390 (10)	0.0426 (11)	−0.0011 (9)	0.0133 (9)	−0.0051 (9)
C3	0.0641 (14)	0.0486 (13)	0.0465 (12)	0.0072 (11)	0.0123 (10)	0.0052 (10)
C4	0.0518 (12)	0.0508 (13)	0.0539 (13)	0.0117 (10)	0.0043 (10)	−0.0034 (11)
C5	0.0389 (10)	0.0487 (12)	0.0525 (12)	−0.0016 (9)	0.0112 (9)	−0.0156 (10)
C6	0.0465 (11)	0.0410 (11)	0.0411 (11)	−0.0061 (9)	0.0122 (9)	−0.0044 (9)
C7	0.0947 (19)	0.0575 (15)	0.0752 (17)	−0.0137 (14)	0.0481 (15)	−0.0009 (13)
O1	0.0598 (9)	0.0502 (9)	0.0562 (9)	0.0115 (8)	0.0108 (7)	0.0016 (7)
N1	0.0526 (10)	0.0451 (10)	0.0522 (11)	−0.0002 (9)	0.0215 (9)	−0.0025 (9)
O2	0.1170 (16)	0.0446 (10)	0.0859 (13)	0.0093 (10)	0.0152 (12)	−0.0144 (10)
O3	0.0709 (11)	0.0675 (11)	0.0518 (10)	0.0030 (8)	0.0038 (8)	−0.0049 (8)

### Geometric parameters (Å, °)

**Table:**

Cl—C5	1.744 (2)	C3—H4	0.9300
N2—C1	1.463 (2)	C4—C5	1.364 (3)
N2—H2	0.8900	C4—H5	0.9300
N2—H1	0.8900	C5—C6	1.388 (3)
N2—H3	0.8900	C6—H6	0.9300
O4—C2	1.349 (2)	C7—H7	0.9600
O4—C7	1.431 (3)	C7—H8	0.9600
C1—C6	1.370 (3)	C7—H9	0.9600
C1—C2	1.397 (3)	O1—N1	1.258 (2)
C2—C3	1.383 (3)	N1—O2	1.228 (2)
C3—C4	1.383 (3)	N1—O3	1.236 (2)
			
C1—N2—H2	109.5	C5—C4—H5	119.9
C1—N2—H1	109.5	C3—C4—H5	119.9
H2—N2—H1	109.5	C4—C5—C6	121.21 (19)
C1—N2—H3	109.5	C4—C5—Cl	120.13 (17)
H2—N2—H3	109.5	C6—C5—Cl	118.66 (17)
H1—N2—H3	109.5	C1—C6—C5	118.04 (19)
C2—O4—C7	118.86 (18)	C1—C6—H6	121.0
C6—C1—C2	122.12 (18)	C5—C6—H6	121.0
C6—C1—N2	120.77 (18)	O4—C7—H7	109.5
C2—C1—N2	117.11 (17)	O4—C7—H8	109.5
O4—C2—C3	126.62 (19)	H7—C7—H8	109.5
O4—C2—C1	115.18 (18)	O4—C7—H9	109.5
C3—C2—C1	118.19 (19)	H7—C7—H9	109.5
C4—C3—C2	120.2 (2)	H8—C7—H9	109.5
C4—C3—H4	119.9	O2—N1—O3	120.9 (2)
C2—C3—H4	119.9	O2—N1—O1	120.0 (2)
C5—C4—C3	120.3 (2)	O3—N1—O1	119.02 (18)
			
C7—O4—C2—C3	12.4 (3)	C2—C3—C4—C5	0.8 (3)
C7—O4—C2—C1	−168.9 (2)	C3—C4—C5—C6	0.1 (3)
C6—C1—C2—O4	−178.65 (18)	C3—C4—C5—Cl	−179.46 (17)
N2—C1—C2—O4	1.4 (3)	C2—C1—C6—C5	0.7 (3)
C6—C1—C2—C3	0.2 (3)	N2—C1—C6—C5	−179.35 (18)
N2—C1—C2—C3	−179.74 (18)	C4—C5—C6—C1	−0.9 (3)
O4—C2—C3—C4	177.7 (2)	Cl—C5—C6—C1	178.71 (15)
C1—C2—C3—C4	−1.0 (3)		

### Hydrogen-bond geometry (Å, °)

**Table:**

*D*—H···*A*	*D*—H	H···*A*	*D*···*A*	*D*—H···*A*
N2—H1···O1^i^	0.89	2.08	2.967 (3)	173
N2—H1···O2^i^	0.89	2.57	3.103 (3)	120
N2—H2···O1	0.89	2.04	2.927 (3)	171
N2—H2···O3	0.89	2.38	3.043 (3)	131
N2—H3···O2^ii^	0.89	2.55	3.240 (3)	135
N2—H3···O3^ii^	0.89	2.07	2.939 (3)	165
C7—H9···O2^iii^	0.96	2.42	3.361 (4)	167

Symmetry codes: (i) *x*, −*y*+1/2, *z*+1/2; (ii) −*x*+2, *y*+1/2, −*z*+3/2; (iii) *x*, *y*+1, *z*.
